# Serum bisphenol A concentration and premature thelarche in female infants aged 4-month to 2-year old

**DOI:** 10.1186/1687-9856-2015-S1-O45

**Published:** 2015-04-28

**Authors:** Lian-hui Chen, Jian-rong Shi, Yan-lan Fang, Li Liang, Wan-qin Chen, Xiao-zhen Chen

**Affiliations:** 1 Department of Pediatrics, The First Affiliated Hospital of College of Medicine, Zhejiang University, Hangzhou, Zhejiang, China; 2 Department of Endocrinology, Children’s Hospital of Zhejiang University School of Medicine, Hangzhou, Zhejiang, China; 3 Zhejiang Quality Inspection Institute of Science, Hangzhou, Zhejiang, China

## Aims

To estimate the association between serum bisphenol A and premature thelarche in female infants aged 4-month to 2-year old.

## Methods

We set a case-control study, a total of 251 female infants (aged 4 months to 2 years) with premature thelarche and 33 healthy age-matched control subjects were enrolled. Test the serum bisphenol A (serum BPA) concentration by liquid-liquid extraction coupled with ultra-performance liquid chromatography tandem mass spectrometry.

## Results

The mean serum BPA levels in the normal and premature thelarche groups were 1.70 ng/ml and 3.48 ng/ml respectively. Serum BPA concentration in the premature thelarche group was significantly greater than that in the control group. There was no correlation between age and serum BPA level. Univariate logistic regression analysis showed that serum BPA concentration positively associated with premature thelarche, and the effect of BPA falls down as the age growing.

## Conclusions

This hospital-based study implies that the association between serum BPA concentrations and premature thelarche. Additionally, the serum BPA levels are much higher than we ever thought in infants, and much more concerns should be raised in infants aged 4-month to 2-year old.

